# Personality Traits in Adolescents with ADHD: Insights into Dimension Evaluation and Clinical Implications Using the Personality Inventory for the DSM-5 Questionnaire

**DOI:** 10.3390/jcm14093048

**Published:** 2025-04-28

**Authors:** Carmela De Domenico, Alessia Fulgenzi, Alessia Andaloro, Marcella Di Cara, Adriana Piccolo, Giulia Marafioti, Fabio Mauro Giambò, Maria Cristina De Cola, Carmela Settimo, Rosalia Muratore, Cecilia Galati, Caterina Impallomeni, Emanuela Tripodi, Francesca Cucinotta

**Affiliations:** 1 IRCCS Centro Neurolesi Bonino Pulejo, S.S. 113 Via Palermo, C. da Casazza, 98124 Messina, Italy; carmela.dedomenico@irccsme.it (C.D.D.); alessia.fulgenzi@irccsme.it (A.F.); andaloro.alessia@libero.it (A.A.); adriana.piccolo@irccsme.it (A.P.); giulia.marafioti@irccsme.it (G.M.); fabio.giambo@irccsme.it (F.M.G.); mariacristina.decola@irccsme.it (M.C.D.C.); carmela.settimo@irccsme.it (C.S.); rosalia.muratore@irccsme.it (R.M.); caterina.impallomeni@irccsme.it (C.I.); emanuela.tripodi@irccsme.it (E.T.); francesca.cucinotta@irccsme.it (F.C.); 2 Department of Health Science, University Magna Graecia, 88100 Catanzaro, Italy; galaticecilia86@gmail.com

**Keywords:** Attention-Deficit/Hyperactivity Disorder, adolescent, personality traits, personalized intervention

## Abstract

**Background/Objectives:** Attention-Deficit/Hyperactivity Disorder (ADHD) is a neurodevelopmental disorder characterized by inattention, hyperactivity, and impulsivity, often persisting into adolescence and adulthood, with significant impacts on social, academic, and occupational functioning. Emerging research highlights the role of personality traits in ADHD, suggesting their influence on symptom presentation, functional impairments, and treatment adherence. This study aims to investigate maladaptive personality domains and traits in adolescents with ADHD using the Alternative Model of Personality Disorders (AMPD) framework and the Personality Inventory for DSM-5 Short Form (PID-5-SF), with a particular focus on the differences among same-sex peers and differences from typically developing peers. **Methods:** This study included 30 ADHD and 25 TD adolescents aged 11–17 (12.6 ± 2.1 vs. 14.9 ± 1.7; *p* < 0.001), recruited at IRCCS Centro Neurolesi “Bonino Pulejo”. Participants underwent clinical assessments, cognitive evaluation, and standardized rating scales, with maladaptive personality traits measured using the PID-5-SF. Chi-squared and Mann–Whitney U tests were used to compare the two groups, as appropriate. **Results:** ADHD adolescents showed significantly higher scores than TD peers in restricted affectivity (*p* = 0.007), distractibility (*p* = 0.001), impulsivity (*p* = 0.049), irresponsibility (*p* = 0.036), hostility (*p* = 0.046), perseveration (*p* = 0.010), submissiveness (*p* = 0.023), and risk-taking (*p* = 0.032). Among personality domains, disinhibition was significantly higher in the ADHD group (*p* = 0.002), while detachment approached significance. Female ADHD participants scored higher than TD females in restricted affectivity, distractibility, and risk-taking. **Conclusions**: These findings suggest that maladaptive personality traits play a key role in ADHD during adolescence, highlighting the need for tailored interventions. Integrating personality assessment into clinical practice may enhance diagnostic accuracy and support more effective, individualized treatment strategies.

## 1. Introduction

Attention-Deficit/Hyperactivity Disorder (ADHD) is a common neurodevelopmental disorder with a global prevalence of approximately 3–5% in childhood [1,2,3,4]. Characterized by inattention, hyperactivity, and impulsivity, ADHD significantly impacts daily functioning and often persists into adolescence and adulthood in a high proportion of cases [5,6,7,8,9,10,11,12]. Longitudinal studies suggest that around 65% of individuals diagnosed in childhood continue to exhibit clinically significant symptoms later in life, with detrimental effects on social and academic functioning and poor socioeconomic outcomes [13,14]. ADHD in late adolescence and adulthood is frequently associated with relational and behavioral challenges, including interpersonal conflicts, higher school dropout rates, substance abuse, and delinquent behaviors. These challenges are linked to lower levels of education, higher unemployment rates, and an increase in failed marriages, crime, and traffic accidents [15,16,17,18,19,20,21,22]. Over the past two decades, research on ADHD has grown significantly. In recent years, the field has evolved from linear, single-cause models to multi-pathway models that highlight ADHD heterogeneity and suggest a link between individual differences at the brain level and clinical presentation [23,24]. Combining genetics, clinical, neurocognitive, and neuroimaging data will help define the functional trajectory of the disorder over time and to identify different responses to treatment. Furthermore, exploring the pathways through which its symptoms and related difficulties evolve may lead to recognizing developmental trajectories associated with ADHD. These trajectories are shaped by a complex interplay of biological, genetic, environmental, and behavioral factors [25]. Furthermore, longitudinal studies have identified three primary developmental trajectories in children with ADHD: a stable trajectory with mild symptoms, an ascending trajectory with worsening symptoms, and a descending trajectory with symptom remission [26,27]. This will be a key step toward personalized and precision approaches to treatment [28]. Among clinical and neurocognitive fields, several studies have focused on analyzing a broad range of neuropsychological domains, such as working memory, inhibition, delay aversion, decision-making [29,30,31], timing, and response variability [32]; however, few studies have formally explored the personality traits of ADHD in adolescence. Personality traits refer to individual habitual patterns of thinking, feeling, and behaving [33] and may be more susceptible to environmental influence [34]. Previous research on the ADHD population shows an association between ADHD and some personality traits (personality dimensions of the integrated five-factor personality model) [35]. More specifically, studies suggest that certain personality traits—such as emotional instability, impulsivity, and distractibility—are significantly linked to ADHD symptomatology. When examined within a maladaptive personality framework, these traits tend to cluster within broader personality domains. Negative affectivity and disinhibition are most strongly related to inattention, while agreeable inhibition and positive emotionality are more strongly associated with impulsivity and hyperactivity [36,37,38]. This a pattern is further confirmed by a recent meta-analytic review [39]. In adolescence, individual differences in personality traits associated with ADHD become even more significant, as this developmental stage is particularly sensitive to such variations in symptomatology [40]. In particular, certain specific personality traits—such as narcissism and callous–unemotional (CU) traits—have been identified as important predictors in differentiating individuals with ADHD from those with other related disorders. Recent studies have shown that narcissistic traits may predict internalizing symptoms, while CU traits are associated with more severe and stable externalizing behaviors, contributing to specific psychopathological trajectories [41,42,43]. However, most of the research available used adult participants. Although the link between ADHD and personality is an intrinsically developmental issue, few studies have examined these links since adolescence. Interestingly, personality traits have emerged as significant predictors of functional impairments, even after accounting for ADHD symptoms, executive function deficits, and current psychiatric comorbidities [44]. Moreover, among the various factors influencing adherence to medication and treatment programs, personality traits play a crucial role in shaping individuals’ perceptions of ADHD, their beliefs about treatment, and their adherence behaviors. In particular, traits such as negative affectivity and antagonism—core dimensions of the Big Five model—or maladaptive facets from the DSM-5 Alternative Model of Personality Disorder, such as intimacy avoidance and deceitfulness, have been found to significantly predict premature treatment discontinuation, beyond other known reasons for non-adherence [45]. Comorbid psychiatric conditions have also been shown to predict poorer outcomes, and specifically, comorbid personality disorder had a significant impact on treatment adherence [46]. All these results support the clinical application of personality assessment in both adult and pediatric age groups with ADHD. However, personality disorders are still underdiagnosed, and categorical diagnosis of personality disorders is not sufficiently clinically useful [47]. The DSM-5 Alternative Model for Personality Disorders (AMPD) provides a dimensional approach to personality pathology [48,49]. The shortcomings of the traditional taxonomy, along with evidence that personality disorders (PDs) are often associated with normal personality traits, have led to a dimensional and empirical approach to understanding individual differences in personality pathology [50]. The AMPD assesses personality pathology in terms of both the severity of personality dysfunction (Criterion A) common to all PDs and the presence of pathological personality domains and traits (Criterion B) [51]. The Personality Inventory for DSM-5 (PID-5) was developed to assess Criterion B of the AMPD. A short form (PID-5-SF) has also been developed [52], an appropriate and useful instrument in research and clinical settings with good psychometric properties [53]. To our knowledge, research on ADHD and dimensional models of personality disorder, particularly in adolescence, remains scarce [54]. This study aims to investigate maladaptive personality domains and traits in ADHD according to AMPD Criterion B using the PID-5-SF. Specifically, we aim to identify the most relevant domains and traits that differentiate ADHD adolescents and their peers with typical development (TD). We, therefore, aim to examine gender-specific variations within the female groups under study. We aim to explore the differences among same-sex peers within the groups studied.

## 2. Materials and Methods

### 2.1. Participants

ADHD and TD adolescents were consecutively recruited at the IRCCS Centro Neurolesi “Bonino Pulejo” in Messina between 2023 and 2024. The enrollment took place after written informed consent was obtained from parents or a legally authorized patient representative. Ethics approval was obtained from the Ethics Committee of the IRCCS Bonino Pulejo of Messina (approval number n.15/2019), and this study was conducted in accordance with the Declaration of Helsinki. Both groups of samples underwent a clinical assessment to verify their fit with the inclusion/exclusion criteria.

### 2.2. Inclusion/Exclusion Criteria

The inclusion criterion for both populations was age between 11 and 17 years. The TD sample did not meet the DSM-5 diagnostic criteria for neurodevelopmental conditions or any other major medical conditions. The inclusion criteria for the ADHD sample were as follows: (a) confirmed ADHD diagnosis, according to DSM-5 criteria, based on a comprehensive clinical and psychological assessment conducted in accordance with the Nice Guideline (Nice, 2019) [55] by experienced psychiatrists; (b) absence of other severe medical conditions (e.g., intellectual disability, severe visual and hearing sensory deficits, head trauma, or other serious genetic disorders); and (c) signed informed consent provided by parents or a legally authorized patient representative. The exclusion criteria were as follows: (a) age outside the range of 11–17 years; (b) failure to meet the diagnostic criteria for ADHD (for the clinical group); (c) presence of other severe medical conditions (e.g., intellectual disability, severe visual and hearing sensory deficits, head trauma, or other serious genetic pathologies); and (d) lack of informed consent from parents or a legally authorized patient representative.

### 2.3. Diagnostic Measures

Diagnostic assessment included a comprehensive clinical and psychosocial evaluation of behavior and symptoms across various domains and settings in daily life, along with a complete developmental and psychiatric history, as well as observer reports and an assessment of the individual’s mental state. Assessment was completed using a cognitive level test—either the Wechsler Intelligence Scale for Children (4th ed.) [56] or Colored Progressive Matrices [57]—and the specific standardized rating scale (Conners, 3rd Edition) [58].

### 2.4. Outcome Measures

The primary outcome measure for this study was the PID-5-SF, 11–17 y.o. This 100-item version of the PID-5-SF was designed to assess maladaptive personality domains and traits in adolescents [59]. The instrument consists of self-report items on a 4-point Likert scale ranging from 0 (very false or usually false) to 3 (very true or usually true). The PID-5-SF assesses five broad domains of personality pathology: negative affectivity, detachment, antagonism, disinhibition, and psychoticism. Additionally, the inventory evaluates a range of maladaptive traits within each of these domains. The negative affectivity domain includes traits such as restricted affectivity, separation insecurity, emotional lability, anxiousness, hostility, submissiveness, and perseveration. The detachment domain includes traits such as withdrawal, intimacy avoidance, depressivity, anhedonia, and suspiciousness. The antagonism domain measures traits such as manipulativeness, grandiosity, deceitfulness, callousness, and attention-seeking, which reflect a tendency toward interpersonal conflict and disregard for the rights of others. The disinhibition domain assesses impulsivity, irresponsibility, rigid perfectionism, distractibility, and risk-taking, capturing traits related to self-control and impulse regulation. The psychoticism domain includes traits such as unusual beliefs and experiences, eccentricity, and unusual perceptual dysregulation, indicative of a more extreme personality style that may be associated with psychosis-like experiences.

### 2.5. Statistical Analysis

Data were analyzed using R software, version 4.3.0, considering a *p*-value < 0.05 as statistically significant. Shapiro–Wilk tests showed that almost all variables followed a non-normal distribution. Therefore, a non-parametric analysis was performed. The Chi-squared test was used to compare proportions, while a Mann–Whitney U test, one- or two-tailed as appropriate, was used to compare the two groups. We used the ‘Rstatix’ package of R to compute Cliff’s delta as a measure of effect size (ES). It is common to interpret effect sizes as “negligible”, “small”, “medium”, or “large” [60,61].

## 3. Results

A total of 60 adolescents took part in the clinical assessment; of these, 5 did not complete the diagnostic interview. Among the remaining n = 55 participants, n = 21 were males and n = 34 females, aged 11 to 17 years. Adolescents were divided into groups based on whether they met the inclusion/exclusion criteria. Of these, 30 were patients diagnosed with ADHD (mean age 12.6 ± 2.1), of which 60% were female. In this group, only n = 4 had co-occurring conditions, specifically Oppositional Defiant Disorder. The remaining 25 constituted a control group composed of TD (mean age 14.9 ± 1.7), with a comparable percentage of female representation. A detailed description of the demographic sample characteristics is reported in Table 1.

After the statistical comparisons of maladaptive personality facet scores between the ADHD and TD patients, the ADHD group presented higher scores than the healthy subjects in almost all personality traits. ADHD vs. TD differences reached statistical significance only in the following subscales: restricted affectivity (*p* < 0.01; effect size= moderate), distractibility (*p* < 0.01; ES = moderate), impulsivity (*p* < 0.05; ES = small), irresponsibility (*p* < 0.05; ES = small), hostility (*p* < 0.05; ES = small), perseveration (*p* < 0.05; ES = moderate; ES = small), submissiveness (*p* < 0.05; ES = small), and risk-taking (*p* < 0.05; ES = small). However, the differences in scores on some subscales were almost significant, such as anhedonia (*p* = 0.07), eccentricity (*p* = 0.08), and rigid perfectionism (*p* = 0.07). Details are provided in Table 2.

Among the maladaptive personality domains (Table 3), the disinhibition pattern shows a significant difference (*p* < 0.01; ES = moderate), while the detachment domain approached significance (*p* = 0.08).

Finally, same-sex peer differences were also assessed. It was found that females in the ADHD group scored significantly higher than females in the TD group on several subscales. In particular, females in the ADHD group had significantly higher scores for the maladaptive personality facets of restricted affectivity (*p* = 0.02), distractibility (*p* = 0.03), and risk-taking (*p* = 0.04).

## 4. Discussion

Our study aimed to assess the presence and differences in maladaptive personality domains and traits between adolescents with ADHD and their TD peers using the PID-5-SF 11–17 y.o. To our knowledge, this is the first study to explore these specific aspects within young adolescents with ADHD through DSM-5-based instruments. The results of this pilot cross-sectional study highlight significant differences in personality traits between adolescents with ADHD and their TD peers, providing valuable insights into the developmental trajectories of ADHD and its long-term implications. Specifically, significant differences in traits such as distractibility, impulsivity, and irresponsibility strongly overlap with the core symptoms of ADHD. These results confirm that the PID-5-SF is sensitive in detecting meaningful differences in personality traits between ADHD and TD. Moreover, they emphasize the pervasive nature of these symptomatic features, which inevitably shape how individuals with ADHD perceive their environment, process information, and behave [62,63]. One of the most striking findings of this study is the significantly higher prevalence of the personality domain of disinhibition in individuals with ADHD. Disinhibition, characterized by poor impulse control, difficulty delaying gratification, and a tendency toward risk-taking, emerges as a defining feature of this clinical population. Specifically, this domain encompasses traits such as impulsivity, irresponsibility, distractibility, and risk-taking, which may contribute to the behavioral instability often observed in individuals with ADHD [64]. Previous research has suggested that personality traits represent relatively stable emotional and behavioral tendencies over time [65,66,67]. Consistent with prior studies, our findings align with the literature indicating a high prevalence of Cluster B personality disorders among individuals with ADHD in young adulthood [68,69] and a retrospective history of ADHD in patients with Borderline Personality Disorder [70]. Furthermore, our study identified differences in personality traits such as submissiveness, restricted affectivity, anhedonia, and rigid perfectionism, delineating a strong component of detachment-related personality domains [71,72]. Not surprisingly, analysis of the data obtained through the PID-5-SF revealed traits associated with Cluster C personality disorders. While these traits do not necessarily constitute a diagnosable personality disorder, they could be conceptualized along a spectrum, offering valuable insights into the psychological profile of individuals with ADHD. These findings are consistent with previous research by Miller et al. [69], which reported an increased prevalence of Cluster C personality disorders (15.8–23.4%) in adulthood. Such differences may reflect individual variations in brain circuits involved in processing and responding to negative stimuli, which play a key role in modulating ADHD symptom severity and long-term outcomes [73,74]. Although biological development, affective context, and sociocultural environment collectively shape personality traits, these elements tend to become deeply embedded and relatively stable over time. Masi et al. (2020) [75] argue that specific behavioral patterns observed in early childhood are closely linked to personality differences in young adulthood and distinct developmental trajectories. Our findings reinforce this perspective, revealing significant differences in personality traits, facets, and domains as early as 12 years of age in individuals with ADHD [76]. The early convergence of ADHD symptomatology with specific personality traits suggests that these individuals may perceive, interpret, and respond to their environment in different ways compared to non-ADHD individuals. The pervasive nature of disinhibition and related traits in ADHD likely influences not only daily functioning but also long-term outcomes in academic, occupational, and social domains [5,77,78,79]. These findings underscore the importance of considering personality dimensions in the assessment and treatment of ADHD [80]. Understanding the interaction between personality traits and ADHD symptoms can enhance diagnostic accuracy and inform more tailored intervention strategies. A neurodevelopmental perspective on personality traits and their early identification, even before adolescence, could help detect atypical developmental trajectories and facilitate the implementation of targeted interventions to prevent negative outcomes in adulthood. Our study also revealed notable differences among same-sex peers. Females with ADHD exhibited significantly higher scores in traits such as distractibility and risk-taking compared to their neurotypical female peers [81,82,83]. These findings align with the previous literature highlighting a higher prevalence of personality disorders within the disinhibition domain. Additionally, ADHD females showed a significantly greater occurrence of reduced affect, a trait typically associated with the detachment personality domain. This trait is often associated with social withdrawal [84], and both elements—more frequently observed in the female ADHD population—have sometimes been described in the literature as potential consequences of impulsive behaviors exhibited by the individuals themselves [85]. This discrepancy may contribute to the underdiagnosis or delayed diagnosis of ADHD in females, with significant implications for timely access to appropriate treatment [86,87]. The interplay between personality traits and developmental trajectories in ADHD has been increasingly explored in recent research. Studies suggest that personality traits may predict the development of co-occurring psychiatric conditions and help distinguish subgroups of patients with different prognoses and intervention needs [88,89,90]. For instance, a narcissistic personality has been linked to an increased risk of internalizing symptoms [91,92], while antisocial and borderline personalities may elevate the risk of severe psychopathologies in adulthood [89,93,94,95]. Identifying these features early may facilitate timely interventions and reduce the likelihood of adverse outcomes [91,96]. While most of the existing literature focuses on the negative impact of personality traits on ADHD outcomes, relatively few studies have examined traits that could serve as protective factors. In light of these findings, a comprehensive and multidimensional approach to ADHD diagnosis and intervention is needed. Identifying specific personality traits and their associations with broader psychosocial outcomes can enable clinicians and researchers to tailor interventions to individual needs. This study supports the use of AMPD taxonomies as valuable tools for assessment in both clinical and research settings. Neurobiological mechanisms, dynamic interaction with environmental factors, and personality traits present since childhood may interact in a complex manner, influencing each other. Dimensional models provide a more accurate representation of personality variation and covariation among traits than traditional categorical frameworks. Their utility extends to both research contexts, where they challenge and refine existing diagnostic criteria for neurodevelopmental disorders, and clinical settings, where they may help predict outcomes and guide targeted interventions.

## 5. Limitations and Future Directions

The PID-5-SF is a useful instrument in research and clinical settings [53] and allows for a multifaceted assessment of personality domains and traits; however, in this study, we did not take into account the pervasiveness and stability of described traits, as well as the level of personality functioning, required for diagnosis of personality disorder. This aspect is acknowledged as a limitation and will need to be addressed through longitudinal research designs in future studies. Nonetheless, our study aimed to explore how this tool may detect personality trait patterns that are specific to ADHD. Future research should examine the stability and pervasiveness of these traits over time, ideally through follow-up studies, to understand their influence on clinical outcomes, comorbidities, and developmental trajectories. Additionally, the PID-5-SF, as a self-report instrument, may introduce response bias or underestimate some dimensions of personality traits. The relatively small sample size is also a limitation, as it may reduce statistical power and limit the generalizability of the findings. Additionally, the sample size did not allow for multivariate analyses or tests of interactions between variables and demographic factors, such as gender. The two groups were not matched for age, with the control group being slightly older than the ADHD group. However, the sample was sufficient to find several significant differences between the two populations to meet the main objective of the research. A notable strength of this study is the high representation of women in the ADHD group, a population that is often underrepresented or misdiagnosed in ADHD research. This inclusion significantly contributes to advancing our understanding of the gender-specific manifestations of the disorder [97]. Finally, it would be interesting to investigate whether traits such as rigid perfectionism, perseveration, and reduced affectivity, when expressed at moderate levels, can positively influence long-term outcomes and mitigate symptom severity. Such analyses could provide valuable insights into personalized interventions and a deeper understanding of the dynamic interaction between personality and ADHD symptomatology.

## 6. Conclusions

This study provides novel evidence of differences in personality traits between adolescents with ADHD and their typically developing peers, contributing to a deeper understanding of the developmental trajectories associated with the disorder. The findings highlight the importance of considering individual and gender-specific factors when designing clinical interventions to promote more adaptive developmental outcomes and prevent psychiatric comorbidity. Clinical implications include the need for more sensitive diagnostic tools and integrated therapeutic approaches that address the different dimensions of emotional and behavioral functioning.

## Figures and Tables

**Table 1:** Demographic description of the two groups.

Characteristics	ADHD	Typical	*p*-Value
N.	30	25	-
Sex			0.98
Male	12 (40.0)	9 (36.0)	
Female	18 (60.0)	16 (64.0)	
Age (years)	12.6 ± 2.1	14.9 ± 1.7	<0.001
Oppositional Defiant Disorder	4	NA	
Conduct Disorder	--	NA	

Continuous variables were expressed as mean ± standard deviation, and *p*-values were calculated using the Mann–Whitney U test, whereas categorical variables (as frequencies and percentages) and *p*-values were calculated using the Chi-square test. NA: not applicable.

**Table 2:** Statistical comparisons of maladaptive personality facet scores between ADHD and TD patients and.

Personality Trait	ADHD Median (Q1–Q3)	Typical Median (Q1–Q3)	ES	*p*-Value
Restricted affectivity	55.0 (48.0–61.2)	45.0 (40.0–52.0)	0.335 (M)	**0.007**
Anhedonia	53.0 (46.0–59.0)	49.0 (38.0–56.0)	0.202 (S)	0.068
Separation insecurity	54.0 (44.0–61.0)	50.0 (37.0–63.0)	0.075 (S)	0.290
Anxiousness	57.5 (48.0–64.2)	56.0 (39.0–62.0)	0.147 (S)	0.140
Unusual beliefs and experiences	51.0 (45.0–57.0)	44.0 (38.0–57.0)	0.175 (S)	0.098
Depressivity	46.0 (42.0–54.2)	42.0 (42.0–52.0)	0.032 (S)	0.409
Perceptual dysregulation	50.5 (41.0–60.2)	49.0 (41.0–58.0)	0.075 (S)	0.292
Distractibility	65.0 (58.0–73.7)	52.0 (42.0–62.0)	0.408 (M)	**0.001**
Eccentricity	49.5 (42.0–61.0)	45.0 (35.0–54.0)	0.189 (S)	0.081
Intimacy avoidance	54.0 (46.0–60.2)	51.0 (46.0–54.0)	0.046 (S)	0.369
Grandiosity	47.0 (39.0–53.0)	50.0 (39.0–56.0)	0.089 (S)	0.748
Impulsivity	54.0 (48.7–66.2)	51.0 (44.0–60.0)	0.224 (S)	**0.049**
Deceitfulness	49.0 (39.7–55.0)	45.0 (38.0–52.0)	0.144 (S)	0.145
Callousness	48.0 (40.0–54.0)	48.0 (40.0–51.0)	0.092 (S)	0.250
Irresponsibility	51.0 (44.0–57.0)	44.0 (44.0–49.0)	0.244 (S)	**0.036**
Emotional lability	47.5 (38.0–58.0)	46.0 (37.0–58.0)	0.021 (S)	0.443
Manipulativeness	49.0 (39.0–52.0)	45.0 (39.0–55.0)	0.048 (S)	0.364
Hostility	58.0 (52.0–64.0)	52.0 (42.0–58.0)	0.228 (S)	**0.046**
Rigid perfectionism	57.0 (48.7–63.0)	41.0 (48.0–60.0)	0.198 (S)	0.072
Perseveration	56.0 (49.0–63.0)	49.0 (41.0–56.0)	0.316 (M)	**0.010**
Attention-seeking	48.0 (42.7–58.2)	48.0 (45.0–59.0)	0.001 (S)	0.507
Withdrawal	53.0 (41.0–60.2)	49.0 (41.0–53.0)	0.171 (S)	0.104
Suspiciousness	56.0 (46.0–65.0)	50.0 (46.0–61.0)	0.158 (S)	0.122
Submissiveness	52.0 (48.0–59.0)	44.0 (37.0–52.0)	0.271 (S)	**0.023**
Risk-taking	51.0 (45.7–63.0)	45.0 (41.0–57.0)	0.251 (S)	**0.032**

Scores are in median (first-third quartile); *p*-values were calculated using the Mann–Whitney U test, and significant differences are in bold. Legend: ES = effect size; S = small; M = medium; L = large.

**Table 3:** Statistical comparisons of clinical scores of personality domains between ADHD patients and typical subjects.

Personality Domain	ADHD Median (Q1–Q3)	Typical Median (Q1–Q3)	ES	*p*-value
Negative affectivity	54.0 (45.2–61.5)	55.0 (33.0–60.0)	0.076 (S)	0.288
Detachment	54.0 (46.7–59.0)	49.0 (44.0–57.0)	0.186 (S)	0.085
Antagonism	48.5 (41.0–53.5)	48.0 (38.0–56.0)	0.095 (S)	0.243
Disinhibition	60.5 (52.0–65.7)	49.0 (44.0–60.0)	0.379 (M)	**0.002**
Psychoticism	51.0 (42.5–59.2)	47.0 (40.0–52.0)	0.170 (S)	0.105

Scores are in median (first-third quartile); *p*-values were calculated using the Mann–Whitney U test, and significant differences are in bold. Legend: ES = effect size; S = small; M = medium; L = large.

## Data Availability

Data and materials related to this study are available upon reasonable request from the corresponding author.

## Abbreviations

The following abbreviations are used in this manuscript:

ADHD	Attention-Deficit/Hyperactivity Disorder
AMPD	Alternative Model of Personality Disorders
CU	Callous–Unemotional
PD	Personality Disorder
PID	Personality Inventory for DSM-5
TD	Typical Development

## References

[1] Polanczyk G., de Lima M.S., Horta B.L., Biederman J., Rohde L.A. (2007). The worldwide prevalence of ADHD: A systematic review and metaregression analysis. Am. J. Psychiatry.

[2] Fayyad J., De Graaf R., Kessler R., Alonso J., Angermeyer M., Demyttenaere K., De Girolamo G., Haro J.M., Karam E.G., Lara C. (2007). Cross-national prevalence and correlates of adult attention-deficit hyperactivity disorder. Br. J. Psychiatry.

[3] Thomas R., Sanders S., Doust J., Beller E., Glasziou P. (2015). Prevalence of attention-deficit/hyperactivity disorder: A systematic review and meta-analysis. Pediatrics.

[4] Posner J., Polanczyk G.V., Sonuga-Barke E. (2020). Attention-deficit hyperactivity disorder. Lancet.

[5] Barkley R.A., Fischer M., Smallish L., Fletcher K. (2002). The persistence of attention-deficit/hyperactivity disorder into young adulthood as a function of reporting source and definition of disorder. J. Abnorm. Psychol..

[6] Rasmussen P., Gillberg C. (2000). Natural outcome of ADHD with developmental coordination disorder at age 22 years: A controlled, longitudinal, community-based study. J. Am. Acad. Child Adolesc. Psychiatry.

[7] Yan W.W. (1998). An investigation of adult outcome of hyperactive children in Shanghai. Psychiatry Clin. Neurosci..

[8] Gittelman R., Mannuzza S. (1985). Hyperactive boys almost grown up. I. Psychiatric status. Arch. Gen. Psychiatry.

[9] Mannuzza S., Klein R.G., Bonagura N., Malloy P., Giampino T.L., Addali K.A. (1991). Hyperactive boys almost grown up. V. Replication of psychiatric status. Arch. Gen. Psychiatry.

[10] Mannuzza S., Klein R.G., Addali K.A. (1991). Young adult mental status of hyperactive boys and their brothers: A prospective follow-up study. J. Am. Acad. Child Adolesc. Psychiatry.

[11] Mannuzza S., Klein R.G., Bessler A., Malloy P., LaPadula M. (1998). Adult psychiatric status of hyperactive boys almost grown up. Am. J. Psychiatry.

[12] Weiss G., Hechtman L., Milroy T., Perlman T. (1985). Psychiatric status of hyperactives as adults: A controlled prospective 15-year follow-up of 63 hyperactive children. J. Child Psychol. Psychiatry.

[13] Faraone S.V., Biederman J. (2005). What is the prevalence of adult ADHD? Results of a population screen of 966 adults. J. Atten. Disord..

[14] Asherson P., Buitelaar J., Faraone S.V., Rohde L.A. (2016). ADHD across the lifespan: Time for change–Practice and research needs to improve the quality of care. Eur. Neuropsychopharmacol..

[15] Barkley R.A., Fischer M., Smallish L., Fletcher K. (2006). Young adult follow-up of hyperactive children: Antisocial activities and drug use. J. Child Psychol. Psychiatry.

[16] DuPaul G.J., Reid R., Anastopoulos A.D., Power T.J. (2014). Assessing ADHD symptomatic behaviors and functional impairment in school settings: Impact of student and teacher characteristics. School Psychol. Q..

[17] Biederman J., Faraone S.V., Spencer T., Wilens T., Norman D., Lapey K.A., Mick E., Lehman B.K., Doyle A. (1993). Patterns of psychiatric comorbidity, cognition, psychosocial functioning in adults with attention deficit hyperactivity disorder. Am. J. Psychiatry.

[18] Kooij J.J., Buitelaar J.K., van den Oord E.J., Furer J.W., Rijnders C.A., Hodiamont P.P. (2005). Internal and external validity of attention-deficit hyperactivity disorder in a population-based sample of adults. Psychol. Med..

[19] Kessler R.C., Adler L., Ames M., Barkley R.A., Birnbaum H., Greenberg P., Johnston J.A., Spencer T., Üstün T.B. (2005). The prevalence and effects of adult attention deficit/hyperactivity disorder on work performance in a nationally representative sample of workers. J. Occup. Environ. Med..

[20] Babinski L.M., Hartsough C.S., Lambert N.M. (1999). Childhood conduct problems, hyperactivity-impulsivity, and inattention as predictors of adult criminal activity. J. Child Psychol. Psychiatry.

[21] Mannuzza S., Klein R.G., Bessler A., Malloy P., LaPadula M. (1993). Adult outcome of hyperactive boys. Educational achievement, occupational rank, and psychiatric status. Arch. Gen. Psychiatry.

[22] Biederman J., Faraone S.V., Spencer T.J., Mick E., Monuteaux M.C., Aleardi M. (2006). Functional impairments in adults with self-reports of diagnosed ADHD: A controlled study of 1001 adults in the community. J. Clin. Psychiatry.

[23] Coghill D.R., Seth S., Matthews K. (2014). A comprehensive assessment of memory, delay aversion, timing, inhibition, decision making and variability in attention deficit hyperactivity disorder: Advancing beyond the three-pathway models. Psychol. Med..

[24] Sonuga-Barke E.J., Coghill D. (2014). Editorial perspective: Laying the foundations for next generation models of ADHD neuropsychology. J. Child Psychol. Psychiatry.

[25] Murray A.L., Russell A., Calderón Alfaro F.A. (2024). Early emotion regulation developmental trajectories and ADHD, internalizing, and conduct problems symptoms in childhood. Dev. Psychopathol..

[26] Breda V., Rohde L.A., Menezes A.M.B., Anselmi L., Caye A., Rovaris D.L., Vitola E.S., Bau C.H.D., Grevet E.H. (2021). The neurodevelopmental nature of attention-deficit hyperactivity disorder in adults. Br. J. Psychiatry.

[27] Wang Y., Ma L., Wang J., Ding Y., Liu N., Men W., Tan S., Gao J.H., Qin S., He Y. (2024). The neural and genetic underpinnings of different developmental trajectories of Attention-Deficit/Hyperactivity Symptoms in children and adolescents. BMC Med..

[28] Cortese S., Coghill D. (2018). Twenty years of research on attention-deficit/hyperactivity disorder (ADHD): Looking back, looking forward. Evid. Based Ment. Health.

[29] Kuntsi J., Oosterlaan J., Stevenson J. (2001). Psychological mechanisms in hyperactivity: I. Response inhibition deficit, working memory impairment, delay aversion, or something else?. J. Child Psychol. Psychiatry.

[30] Sonuga-Barke E., Bitsakou P., Thompson M. (2010). Beyond the dual pathway model: Evidence for the dissociation of timing, inhibitory, and delay-related impairments in attention-deficit/hyperactivity disorder. J. Am. Acad. Child Adolesc. Psychiatry.

[31] Solanto M.V., Abikoff H., Sonuga-Barke E., Schachar R., Logan G.D., Wigal T., Hechtman L., Hinshaw S., Turkel E. (2001). The ecological validity of delay aversion and response inhibition as measures of impulsivity in ADHD: A supplement to the NIMH multimodal treatment study of AD/HD. J. Abnorm. Child Psychol..

[32] Fair D.A., Bathula D., Nikolas M.A., Nigg J.T. (2012). Distinct neuropsychological subgroups in typically developing youth inform heterogeneity in children with ADHD. Proc. Natl. Acad. Sci. USA.

[33] John O.P., John O.P., Robins R.W. (2021). History, measurement, and conceptual elaboration of the Big-Five trait taxonomy. Handbook of Personality: Theory and Research.

[34] Srivastava S., John O.P., Gosling S.D., Potter J. (2003). Development of personality in early and middle adulthood: Set like plaster or persistent change?. J. Personal. Soc. Psychol..

[35] Markon K.E., Krueger R.F., Watson D. (2005). Delineating the structure of normal and abnormal personality: An integrative hierarchical approach. J. Personal. Soc. Psychol..

[36] Gomez R., Corr P.J. (2014). ADHD and personality: A meta-analytic review. Clin. Psychol. Rev..

[37] Nigg J.T. (2006). Temperament and developmental psychopathology. J. Child Psychol. Psychiatry.

[38] Knouse L.E., Traeger L., O’Cleirigh C., Safren S.A. (2013). Adult attention deficit hyperactivity disorder symptoms and five-factor model traits in a clinical sample: A structural equation modeling approach. J. Nerv. Ment. Dis..

[39] Jacobsson P., Hopwood C.J., Söderpalm B., Nilsson T. (2021). Adult ADHD and emerging models of maladaptive personality: A meta-analytic review. BMC Psychiatry.

[40] Bell L.J., John O.P., Hinshaw S.P. (2024). ADHD Symptoms in Childhood and Big Five Personality Traits in Adolescence: A Five-Year Longitudinal Study in Girls. Res. Child Adolesc. Psychopathol..

[41] Masi G., Muratori P., Manfredi A., Pisano S., Milone A. (2015). Child behaviour checklist emotional dysregulation profiles in youth with disruptive behaviour disorders: Clinical correlates and treatment implications. Psychiatry Res..

[42] Muratori P., Milone A., Levantini V., Pisano S., Spensieri V., Valente E., Thomaes S., Masi G. (2020). Narcissistic traits as predictors of emotional problems in children with oppositional defiant disorder: A longitudinal study. J. Affect. Disord..

[43] Faraone S.V., Rostain A.L., Blader J., Busch B., Childress A.C., Connor D.F., Newcorn J.H. (2019). Practitioner Review: Emotional dysregulation in attention-deficit/hyperactivity disorder–implications for clinical recognition and intervention. J. Child Psychol. Psychiatry Allied Discip..

[44] He J.A., Antshel K.M., Biederman J., Faraone S.V. (2019). Do Personality Traits Predict Functional Impairment and Quality of Life in Adult ADHD? A Controlled Study. J. Atten. Disord..

[45] Jacobsson P., Granqvist T., Hopwood C.J., Krueger R.F., Söderpalm B., Nilsson T. (2025). How Do Personality Dysfunction and Maladaptive Personality Traits Predict Time to Premature Discontinuation of Pharmacological Treatment of ADHD?. J. Atten. Disord..

[46] Lensing M.B., Zeiner P., Sandvik L., Opjordsmoen S. (2013). Four-Year Outcome in Psychopharmacologically Treated Adults with Attention-Deficit/Hyperactivity Disorder: A Questionnaire Survey. J. Clin. Psychiatry.

[47] Morey L.C., Benson K.T. (2016). An Investigation of Adherence to Diagnostic Criteria, Revisited: Clinical Diagnosis of the DSM-IV/DSM-5 Section II Personality Disorders. J. Personal. Disord..

[48] American Psychiatric Association (2022). Diagnostic and Statistical Manual of Mental Disorders.

[49] Zimmermann J., Kerber A., Rek K., Hopwood C.J., Krueger R.F. (2019). A Brief but Comprehensive Review of Research on the Alternative DSM-5 Model for Personality Disorders. Curr. Psychiatry Rep..

[50] Karalunas S.L., Gustafsson H.C., Fair D.A. (2022). Dimensional Approaches to ADHD: Implications for Research and Clinical Practice. Curr. Opin. Psychol..

[51] Krueger R.F., Derringer J., Markon K.E., Watson D., Skodol A.E. (2012). Initial construction of a maladaptive personality trait model and inventory for DSM-5. Psychol. Med..

[52] Maples J.L., Carter N.T., Few L.R., Crego C., Gore W.L., Samuel D.B., Williamson R.L., Lynam D.R., Widiger T.A., Markon K.E. (2015). Testing Whether the DSM-5 Personality Disorder Trait Model Can Be Measured with a Reduced Set of Items: An Item Response Theory Investigation of the Personality Inventory for DSM-5. Psychol. Assess..

[53] Thimm J.C., Jordan S., Bach B. (2016). The Personality Inventory for DSM-5 Short Form (PID-5-SF): Psychometric Properties and Association with Big Five Traits and Pathological Beliefs in a Norwegian Population. BMC Psychol..

[54] Jacobsson P., Hopwood C.J., Krueger R.F., Söderpalm B., Nilsson T. (2024). Conceptualizing Adult ADHD with the DSM Alternative Model of Personality Disorder. Personal. Ment. Health.

[55] National Institute for Health and Care Excellence (NICE) (2019). Surgical Site Infections: Prevention and Treatment. NICE Guideline [NG125].

[56] Wechsler D. (2003). Wechsler Intelligence Scale for Children—Fourth Edition (WISC-IV).

[57] Raven J.C. (1962). Coloured Progressive Matrices.

[58] Conners C.K. (2008). Conners 3rd Edition Manual.

[59] Fossati A., Borroni S., Del Corno F., Krueger R.F., Markon K.E. (2015). Inventario di Personalità per il DSM-5—Short Form (PID-5-SF)—Soggetto 11-17 Anni.

[60] Cliff N. (1996). Ordinal Methods for Behavioral Data Analysis.

[61] Cohen J. (1988). Statistical Power Analysis for the Behavioral Sciences.

[62] Becker S.P. (2013). The Internal, External, and Diagnostic Validity of Sluggish Cognitive Tempo: A Meta-Analysis and Critical Review. J. Am. Acad. Child Adolesc. Psychiatry.

[63] Ashinoff B.K., Abu-Akel A. (2021). Hyperfocus: The forgotten frontier of attention. Psychol. Res..

[64] Gomez R., Watson S., Brown T. (2021). Inter-relationships Between ADHD, ODD and Impulsivity Dimensions in Emerging Adults: A Network Analysis. Heliyon.

[65] Battaglia M., Przybeck T.R., Bellodi L., Cloninger C.R. (1996). Temperament dimensions explain the comorbidity of psychiatric disorders. Compr. Psychiatry.

[66] Cloninger C.R., Martin R.L., Guze S.B., Clayton P.J., Maser J.D., Cloninger C.R. (1990). The empirical structure of psychiatric comorbidity and its theoretical significance. Comorbidity of Mood and Anxiety Disorders.

[67] Crawford T.N., Cohen P., First M.B., Skodol A.E., Johnson J.G., Kasen S. (2008). Comorbid Axis I and Axis II disorders in early adolescence: Outcomes 20 years later. Arch. Gen. Psychiatry.

[68] Halperin J.M., Rucklidge J.J., Powers R.L., Miller C.J., Newcorn J.H. (2011). Childhood CBCL bipolar profile and adolescent/young adult personality disorders: A 9-year follow-up. J. Affect. Disord..

[69] Miller T.W., Nigg J.T., Faraone S.V. (2007). Axis I and II comorbidity in adults with ADHD. J. Abnorm. Psychol..

[70] Fossati A., Novella L., Donati D., Donini M., Maffei C. (2002). History of childhood attention deficit/hyperactivity disorder symptoms and borderline personality disorder: A controlled study. Compr. Psychiatry.

[71] Sireli O., Colak M., Demirci T.H., Savascihabes A.E., Oz Cinar H. (2024). Early Maladaptive Schemas in Adolescents with Attention Deficit Hyperactivity Disorder. Front. Psychiatry.

[72] Gomez R., Brown T. (2024). Incremental Validity of ADHD Dimensions in the Predictions of Emotional Symptoms, Conduct Problems, and Peer Problems in Adolescents Based on Parent, Teacher, and Self-Ratings. Pediatr. Rep..

[73] Gutiérrez F., Valdesoiro F. (2023). The evolution of personality disorders: A review of proposals. Front. Psychiatry.

[74] Michelini G., Kitsune G.L., Cheung C.H.M., Brandeis D., Banaschewski T., Asherson P., Rubia K., Kuntsi J. (2021). Attention-Deficit/Hyperactivity Disorder Remission Is Linked to Better Neurophysiological Error Detection and Attention-Vigilance Processes. Eur. Psychiatry.

[75] Masi G., Fantozzi P., Muratori P., Bertolucci G., Tacchi A., Villafranca A., Pfanner C., Cortese S. (2020). Emotional dysregulation and callous unemotional traits as possible predictors of short-term response to methylphenidate monotherapy in drug-naïve youth with ADHD. Compr. Psychiatry.

[76] Nigg J.T., Sibley M.H., Thapar A., Karalunas S.L. (2020). Development of ADHD: Etiology, heterogeneity, and early life course. Annu. Rev. Dev. Psychol..

[77] Klein R.G., Mannuzza S., Olazagasti M.A., Roizen E., Hutchison J.A., Castellanos F.X. (2012). Clinical and functional outcome of childhood ADHD 33 years later. Arch. Gen. Psychiatry.

[78] Biederman J., Petty C.R., Evans M., Small J., Faraone S.V. (2010). How persistent is ADHD? A controlled 10-year follow-up study of boys with ADHD. Psychol. Med..

[79] Vize C.E., Byrd A.L., Stepp S.D. (2023). The Relative Importance of Psychopathy Features as Predictors of Externalizing Behaviors in Youth: A Multimethod Examination. J. Psychopathol. Behav. Assess..

[80] Molina B.S.G., Hinshaw S.P., Swanson J.M., Arnold L.E., Hechtman L., Jensen P.S. (2023). ADHD Symptom Persistence from Childhood Through Adolescence and Emerging Adulthood: A 10-Year Follow-Up Study. J. Child Psychol. Psychiatry.

[81] Koutsoklenis A., Honkasilta J. (2023). ADHD in the DSM-5-TR: What has changed and what has not. Front. Psychiatry.

[82] Gilbert M., Banaschewski T., Buitelaar J.K., Coghill D.R., Dittmann R.W., Hollis C., Döpfner M. (2023). Gender and Age Differences in ADHD Symptoms and Co-Occurring Depression and Anxiety Symptoms Among Children and Adolescents. Child Psychiatry Hum. Dev..

[83] Vine V., Byrd A.L., Mohr H., Stepp S.D. (2020). The Structure of Psychopathology in a Sample of Clinically Referred, Emotionally Dysregulated Early Adolescents. J. Abnorm. Child Psychol..

[84] Young S., Adamo N., Ásgeirsdóttir B.B., Branney P., Beckett M., Colley W., Cubbin S., Deeley Q., Farrag E., Gudjonsson G. (2020). Females with ADHD: An expert consensus statement taking a lifespan approach providing guidance for the assessment and treatment of attention-deficit hyperactivity disorder in girls and women. BMC Psychiatry.

[85] Harpin V., Mazzone L., Raynaud J.P., Kahle J., Hodgkins P. (2016). Long-term outcomes of ADHD: A systematic review of self-esteem and social function. J. Atten. Disord..

[86] Hinshaw S.P., Nguyen P.T., O’Grady S.M., Rosenthal E.A. (2022). Annual Research Review: Attention-deficit/hyperactivity disorder in girls and women: Underrepresentation, longitudinal processes, and key directions. J. Child Psychol. Psychiatry.

[87] Hasson R., Fine J.G. (2012). Gender differences among children with ADHD on continuous performance tests: A meta-analytic review. J. Atten. Disord..

[88] Masi G., Milone A., Brovedani P., Pisano S., Muratori P. (2018). Psychiatric evaluation of youths with disruptive behavior disorders and psychopathic traits. Neurosci. Biobehav. Rev..

[89] Pardini D.A., Loeber R. (2008). Interpersonal callousness trajectories across adolescence: Early social influences and adult outcomes. Crim. Justice Behav..

[90] Ginsberg Y., Hirvikoski T., Lindefors N. (2010). Attention Deficit Hyperactivity Disorder (ADHD) among longer-term prison inmates is a prevalent, persistent and disabling disorder. BMC Psychiatry.

[91] Thomaes S., Stegge H., Bushman B.J., Olthof T., Denissen J. (2008). Development and validation of the Childhood Narcissism Scale. J. Personal. Assess..

[92] Washburn J.J., McMahon S.D., King C.A., Reinecke M.A., Silver C. (2004). Narcissistic features in young adolescents: Relations to aggression and internalizing symptoms. J. Youth Adolesc..

[93] McMahon R.J., Witkiewitz K., Kotler J.S., Conduct Problems Prevention Research Group (2010). Predictive validity of callous-unemotional traits measured in early adolescence with respect to multiple antisocial outcomes. J. Abnorm. Psychol..

[94] Stepp S.D., Lazarus S.A., Byrd A.L. (2011). Reciprocal influences between parents’ mental health symptoms and children’s behavior problems: Bidirectional associations from preschool to adolescence. J. Abnorm. Child Psychol..

[95] Matthies S.D., Philipsen A. (2014). Common ground in Attention Deficit Hyperactivity Disorder (ADHD) and Borderline Personality Disorder (BPD)—Review of recent findings. Borderline Personal. Disord. Emot. Dysregul..

[96] Beauchaine T.P., Gatzke-Kopp L., Mead H.K. (2007). Polyvagal theory and developmental psychopathology: Emotion dysregulation and conduct problems from preschool to adolescence. Biol. Psychol..

[97] Faraone S.V., Banaschewski T., Coghill D., Zheng Y., Biederman J., Bellgrove M.A., Newcorn J.H., Gignac M., Al Saud N.M., Manor I. (2021). The World Federation of ADHD International Consensus Statement: 208 evidence-based conclusions about the disorder. Neurosci. Biobehav. Rev..

